# Establishment of a Visual LAMP Technology and Detection of *Cronartium ribicola* Infecting Chinese White Pine in Southwestern China

**DOI:** 10.3390/jof12060409

**Published:** 2026-06-04

**Authors:** Xinyi Zhang, Zijia Peng, Ruonan Jing, Xinye Liu, Tauseef Ullah, Min Sheng, Zhongdong Yu

**Affiliations:** Key Laboratory of National Forestry and Grassland Administration on Management of Western Forest Bio-Disaster, College of Forestry, Northwest A&F University, Yangling 712100, China; zhangxinyi1@nwafu.edu.cn (X.Z.); jeremy0704@nwafu.edu.cn (Z.P.); 2024060432@nwafu.edu.cn (R.J.); line0524@126.com (X.L.); tauseefullah03@nwafu.edu.cn (T.U.); shengmin1977@126.com (M.S.)

**Keywords:** *Pinus armandii*, *Cronartium ribicola*, loop-mediated isothermal amplification (LAMP), hydroxynaphthol blue (HNB), global climate change

## Abstract

White pine blister rust disease (WPBR), caused by *Cronartium ribicola*, ranks among the most destructive pathogens of five-needle pines. We developed a hydroxynaphthol blue (HNB)-based Loop-mediated isothermal amplification (LAMP) assay enabling rapid, visual detection of *C. ribicola* directly following DNA extraction. LAMP primers targeting the internal transcribed spacer (ITS) region were designed and validated through in silico comparison with related *Cronartium* species and in vitro testing against sympatric forest fungi. The optimized 25 μL reaction contained 8.0 mM Mg^2+^, 1.0 mM dNTPs, and an inner-to-outer primer ratio of 8:1, with amplification conducted at 62 °C for 40 min. Positive amplification produced a distinctive color transition from purple to sky blue, enabling visual interpretation without instrumentation. Under the tested conditions, the assay achieved a detection limit of 460 ± 3.2 fg/μL genomic DNA—a 10-fold improvement over conventional PCR in concentration-based sensitivity. Assay applicability was evaluated using 211 field-collected *Pinus armandii* samples sourced from China. Detection efficiency varied significantly across tissue types. Symptomatic bark exhibited a substantially higher positive detection rate (68.97%, 95% CI: 49.2–84.7%) compared to needles from symptomatic trees (18.75%, 95% CI: 4.1–45.7%). Among asymptomatic samples, 3.75% of bark samples tested positive for *C. ribicola* DNA, whereas all needle samples were negative. Geographically, positive detections clustered at several discrete sampling sites in southwestern China, predominantly at elevated elevations. The established LAMP-HNB assay provides a rapid, visually interpretable diagnostic tool for early detection and quarantine monitoring of WPBR following DNA extraction. Beyond its practical utility, this assay establishes valuable baseline data for targeted disease surveillance in the context of evolving climate conditions.

## 1. Introduction

White pine blister rust (WPBR) is a devastating disease caused by *Cronartium ribicola* J. C. Fischer, posing a serious threat to global pine forest resources. The disease originated in Asia and subsequently became prevalent in Europe and North America [1], exhibiting strong ecological adaptability and destructive potential [2,3]. In North America, WPBR causes seedling mortality rates exceeding 95% in western white pine (*Pinus monticola*) and inflicts severe economic and ecological losses across other five-needle pine species, including sugar pine (*P. lambertiana*), eastern white pine (*P. strobus*), whitebark pine (*P. albicaulis*), and limber pine (*P. flexilis*) [4,5]. In China, WPBR primarily affects important tree species such as Korean pine (*P. koraiensis*) and Chinese white pine (*P. armandii*). Its distribution covers multiple regions, including Northeast China, the Hengduan and Himalayan Mountains in Southwest China, and the Qinling–Daba Mountains [6], posing a long-term threat to forest health and biosecurity in China. However, an unexpected phenomenon has emerged under changing global climatic conditions: WPBR incidence in regions historically identified as disease hotspots has declined relative to observations from two decades ago. At several sites, *C. ribicola* remains undetected despite the presence of multi-aged white pine stands, raising uncertainty as to whether these pines are genuinely disease-free or harbor latent infections. We hypothesize that the pathogen persists in a latent state within pine tissues for extended periods without producing visible symptoms under altered climate conditions and that current detection failures stem from the absence of sufficiently sensitive diagnostic technologies.

*C. ribicola* has a macrocyclic and heteroecious life cycle, completing its infection cycle primarily between five-needle pines and alternate host plants from the genera *Ribes*, *Pedicularis*, and *Castilleja* [4,7]. It sequentially produces five different spore types: spermatia, aeciospores, urediniospores, teliospores, and basidiospores. Among these, spermatia and aeciospores are produced mainly on five-needle pines, while urediniospores, teliospores, and basidiospores develop on the lower leaf surfaces of alternate hosts, such as *Ribes* spp., within Chinese white pine forests [8]. Basidiospores typically infect the needles in late summer, and the infection hyphae subsequently migrate to the trunk bark after one or two years, depending on host resistance and local climatic conditions [9,10]. Due to its obligate parasitism, complex life cycle, long latent period, atypical early symptoms, and genetically specific interactions with various five-needle pine hosts, the rapid detection and management of this pathogen remain highly challenging. Traditional identification methods based on morphological and pathological characteristics are constrained by prolonged processing times, limited sensitivity, and inherent subjectivity [8]. Current quarantine and diagnostic methods for WPBR primarily rely on symptom observation, tissue staining, and quantitative PCR detection. However, symptom diagnosis is constrained by the seasonal occurrence of the disease, while histological tissue staining and metabolite analyses are operationally complex and require specialized equipment [11,12], hindering rapid, convenient, and accurate field diagnosis. In recent years, molecular diagnostic technologies have advanced rapidly, establishing nucleic acid-based methods as the standard approach for early, accurate diagnosis and effective management of forest diseases [13]. Among these approaches, Loop-mediated isothermal amplification (LAMP) is particularly well-suited for rapid pathogen detection in field settings and primary forestry stations, owing to its operational simplicity, fast usage, high sensitivity and specificity, and isothermal amplification conditions that eliminate the need for specialized thermal cycling equipment. Consequently, developing an efficient, sensitive, and highly specific molecular detection method featuring simplified amplification and visual readout following DNA extraction holds substantial significance for field-based monitoring, early warning systems, and integrated management of WPBR.

Loop-mediated isothermal AMPlification (LAMP) is a nucleic acid isothermal amplification method developed by Notomi in 2000 [14]. This technology relies on 4–6 specific primers and Bst DNA polymerase with strand displacement activity, enabling amplification of the target sequence by over 10^9^ times within 30–60 min under isothermal conditions (typically 60–65 °C) [14]. Compared to conventional PCR, LAMP technology offers advantages such as simple operation, rapid reaction, high sensitivity, and strong specificity [15]. Furthermore, results can be interpreted visually through closed-tube detection methods like turbidimetry or colorimetry, effectively avoiding aerosol contamination. This makes it particularly suitable for rapid detection scenarios in customs quarantine, resource-limited settings, and field environments. In recent years, LAMP technology has been widely applied across medical, food, animal husbandry, and plant pathology diagnostics [16,17,18,19], demonstrating robust technical applicability and substantial promise. Recent studies have made significant progress in molecular field diagnostics for *C. ribicola*. Capron et al. developed an in situ processing and efficient environmental detection (iSPEED) system utilizing point-of-use real-time PCR [11], while Kozhar et al. established a rapid LAMP-based colorimetric assay for white pine blister rust [20]. Building on these foundational developments, this study aimed to optimize a visual LAMP-HNB assay and evaluate its performance on *P. armandii* samples collected from high-altitude regions in southwestern China.

This study focused on developing a visual LAMP assay for *C. ribicola* and applying it to investigate the distribution pattern of WPBR in China. Specific LAMP primers were designed to target the conserved internal transcribed spacer (ITS) region of the ribosomal DNA. Through systematic primer screening, optimization of the reaction conditions, and sensitivity evaluation, a visual LAMP detection method was established using hydroxynaphthol blue (HNB) dye [21]. The overarching objective was to establish a rapid, sensitive, and visually interpretable molecular detection platform for *C. ribicola* that would enable early diagnosis, support epidemiological surveillance, and inform evidence-based management strategies for WPBR in China.

## 2. Materials and Methods

### 2.1. Materials

The analytical specificity panel comprised six fungal species commonly encountered in *Pinus armandii* forests: *Cronartium ribicola*, *Melampsora larici-populina*, *Gymnosporangium asiaticum*, *Sawadaea tulasnei*, *Erysiphe paeoniae*, and *Flammulina velutipes*. *C. ribicola* isolates were obtained from diseased *P. armandii* bark collected in Butuo County (Sichuan Province) and from diseased *P. koraiensis* bark collected in Benxi County (Liaoning Province). Reference strains of the remaining fungi were maintained at the Forest Pathology Laboratory, Northwest A&F University (Yangling, China). All strains were used exclusively to assess analytical specificity within the defined test panel.

A total of 211 *P. armandii* samples, encompassing symptomatic and asymptomatic needles, bark, and occasional root tissues, were collected from multiple forest stations across Sichuan, Yunnan, Shaanxi, and Gansu provinces between October 2023 and May 2025 (Table 1). The sample collection was conducted in three temporal cohorts: (i) 60 asymptomatic needle and bark samples collected in October 2023; (ii) 73 samples (including asymptomatic and symptomatic needles, bark, and roots) collected in April 2024; and (iii) 78 samples (including both asymptomatic and symptomatic needles and bark) collected in May 2025. Detailed sampling metadata, including geographic coordinates, host species, altitude range, and tissue type, are presented in Table 1. Samples were collected at the tissue-sample level rather than as a fully paired tree-level design. When feasible, bark and needle tissues were obtained from the same individual; however, paired bark–needle sampling was not consistently achieved across all trees, resulting in unequal bark and needle sample numbers among sites and disease-status categories. Consequently, tissue-type comparisons were performed at the sample level.

Sampling locations were georeferenced as follows: Butuo (102.82° E, 27.71° N), Maoxian (103.86° E, 31.69° N), Jinyang (103.25° E, 27.70° N), Kangding (101.96° E, 30.04° N), Luding (102.24° E, 29.92° N), Nanjiang (106.83° E, 32.35° N), Zhaojue (102.85° E, 28.02° N), Meigu (103.14° E, 28.33° N), Danba (101.90° E, 30.88° N), Jiangyou (104.75° E, 31.78° N), and Huidong (102.58° E, 26.64° N) in Sichuan Province; Qiaojia (102.94° E, 26.91° N) in Yunnan Province; Liuba (106.93° E, 33.62° N), Fengxian (106.52° E, 33.92° N), Huayin (110.10° E, 34.57° N), and Zhouzhi (108.23° E, 34.17° N) in Shaanxi Province; and Liangdang (106.55° E, 34.14° N) in Gansu Province. In parallel, two *C. ribicola* aeciospore samples were used as positive controls: CR1, collected from *P. armandii* in Butuo, and CR2, collected from *P. koraiensis* in Benxi (125.30° E, 41.35° N). Double-distilled water (ddH_2_O) was used as a negative control.

Primers were synthesized by Sangon Biotech Co., Ltd. (Shanghai, China). Bst 3.0 DNA polymerase was obtained from New England Biolabs (NEB, Ipswich, MA, USA). The dNTP Mix (10 mM) and hydroxynaphthol blue (HNB, 100×) reagents were purchased from Beijing Solarbio Science & Technology Co., Ltd. (Beijing, China).

### 2.2. Genomic DNA Extraction

DNA extraction was performed uniformly across all sample types using a modified cetyltrimethylammonium bromide (CTAB) protocol, including fungal pure cultures and various *P. armandii* tissues (needles, bark, and roots). Approximately 5 mg of each sample was weighed, ground into a fine powder using liquid nitrogen, and quickly transferred into a pre-chilled 1.5 mL centrifuge tube. Subsequently, 1 mL of CTAB pre-treatment buffer (0.2 mol/L Tris-HCl, 0.25 mol/L NaCl, 0.05 mol/L EDTA) was added, mixed uniformly, and incubated on ice for 10 min, followed by centrifugation at 4 °C and 10,000 rpm for 10 min. The supernatant was discarded. Pre-warmed 2× CTAB extraction buffer (650 μL; containing 20 g/L CTAB, 0.1 mol/L Tris-HCl, 1.5 mol/L NaCl, 0.05 mol/L EDTA) and β-mercaptoethanol (2 μL) were added to the pellet. The mixture was incubated at 65 °C for 1 h with gentle inversion at 10 min intervals. Following incubation, an equal volume (500 μL) of DNA extraction solution (chloroform:isoamyl alcohol, 24:1) was added, gently mixed for 10 min, and centrifuged at room temperature (11,000× *g*, 10 min). This extraction step was repeated twice. The resulting supernatant was collected, mixed with 500 μL of pre-cooled isopropanol by gentle inversion, and stored at −20 °C for 4 h in order to precipitate the DNA. Following centrifugation at room temperature at 11,000 rpm for 10 min, the supernatant was discarded. The pellet was washed twice with 600 μL of 75% ethanol and centrifuged at 12,000 rpm for 10 min at room temperature. Finally, the pellet was air-dried in a clean bench, dissolved in 50 μL of TE buffer, and stored at −20 °C for subsequent use.

### 2.3. LAMP Primer Design and Screening

The genomic DNA of *C. ribicola* was used for ITS sequence amplification. Following sequencing and assembly, the conserved ITS sequence of *C. ribicola* was obtained as the target region. Based on the ITS sequence, LAMP primers for *C. ribicola* were designed using the online software Primer Explorer V5 (http://primerexplorer.jp/e/, accessed on 1 May 2025). Two candidate primer sets were initially selected based on dimer values and the terminal free energy. Each set comprised two inner primers (FIP and BIP) and two outer primers (F3 and B3), as detailed in Table 2. All primers were synthesized by Sangon Biotech Co., Ltd. (Shanghai, China).

To evaluate the interspecific specificity and genomic uniqueness of the designed primers, an in silico analysis was performed. Internal transcribed spacer (ITS) sequences were retrieved from the NCBI GenBank database for *C. ribicola* (EU826970.1) and four closely related non-target species: *C. comandrae* (JN943211.1), *C. comptoniae* (JN943208.1), *C. quercuum* (JN943193.1), and *C. strobilinum* (JN943191.1). Multiple sequence alignment was conducted using SnapGene v8.2.2 to identify species-specific single-nucleotide polymorphisms (SNPs) within the primer binding regions of the candidate primer sets.

### 2.4. Primer Specificity Test

An initial LAMP reaction system was established following the instructions of the Bst 3.0 DNA polymerase kit (NEB, Ipswich, MA, USA), as outlined in Table 3. LAMP was evaluated using genomic DNA extracted from *C. ribicola*, *M. larici-populina*, *G. asiaticum*, *E. paeoniae*, *S. tulasnei*, and *F. velutipes* as templates. All reaction components were assembled in PCR tubes on ice and mixed by short vortexing. The initial LAMP reaction was performed in a water bath at 65 °C for 60 min, followed by enzyme inactivation at 80 °C for 10 min. After the reaction, 3 μL of the amplification product was analyzed by 1.5% agarose gel electrophoresis. Primer sets that successfully amplified only *C. ribicola* genomic DNA, as confirmed by the presence of characteristic ladder-like banding patterns on the agarose gel, were selected for subsequent specificity testing and optimization assays.

### 2.5. Optimization of Reaction System and Conditions

Based on the initial reaction system and conditions, single-factor optimization was performed sequentially for Mg^2+^ concentration, dNTP concentration, the ratio of inner to outer primers, temperature, and reaction time, according to the gradients specified in Table 4. Double-distilled water (ddH_2_O) was used as a blank negative control. After the reaction, 3 μL of the amplification product was analyzed by 1.5% agarose gel electrophoresis. The optimal concentration and reaction conditions were determined based on the presence and clarity of the typical LAMP ladder-like bands. For visual detection, hydroxynaphthol blue (HNB; 10× concentration) was added (1.5 μL per reaction) to indicate LAMP results. All assays were performed in triplicate. Results were visually assessed based on the color shift in the HNB indicator, with positive reactions displaying a sky blue coloration and negative reactions remaining purple. Assessments were performed using positive and negative controls as references.

### 2.6. Sensitivity Detection Methods

The concentration of the extracted *C. ribicola* genomic DNA was quantified and then serially diluted 10-fold to prepare templates of varying concentrations. LAMP reactions were performed using the optimized system and conditions, with ddH_2_O serving as a blank control. The lowest detectable concentration (detection limit) was determined by observing the color change in the HNB dye after the reaction.

Concurrently, conventional PCR was performed using the universal fungal primers ITS1 and ITS4 for comparison [22]. The total PCR reaction volume was 25 μL, consisting of 12.5 μL of PCR Master Mix, 1.5 μL each of the forward and reverse primers (10 μM), 3 μL of template DNA, and ultra-pure water up to 25 μL. The thermal cycling conditions were as follows: initial denaturation at 94 °C for 5 min; 35 cycles of denaturation at 94 °C for 30 s, annealing at 58 °C for 30 s, and extension at 72 °C for 1 min; followed by a final extension at 72 °C for 10 min. After amplification, 3 μL of the PCR product was analyzed by 1.5% agarose gel electrophoresis to determine the detection limit of conventional PCR for *C. ribicola*. Finally, the analytical sensitivity of the visual LAMP assay was compared with that of conventional PCR. Serial dilutions of template DNA (each performed in triplicate) were subjected to both assays. Template DNA concentrations were quantified using a spectrophotometer (NanoDrop 2000C, Thermo Fisher Scientific, Waltham, MA, USA), and detection limits were determined based on mean ± standard deviation (SD) values across replicates.

### 2.7. Statistical Analysis

Detection data were analyzed using a combination of descriptive and inferential statistical methods. Positive detection rates were calculated for samples stratified by tissue type (bark, needles, and roots) and geographic origin, with results expressed as percentages. The 95% confidence intervals (CIs) for positive detection proportions were calculated using the Clopper–Pearson exact method. To assess the effect of tissue type on detection outcomes, a chi-square (χ^2^) test was employed to compare positive rates between diseased bark and diseased needles; results were further verified using Fisher’s exact test where appropriate. All statistical analyses were performed using SPSS 26.0 software (IBM Corp., Armonk, NY, USA), with the significance level set at α = 0.05. Additionally, the geographic distribution of positive samples relative to altitude was characterized to elucidate the spatial distribution pattern of *C. ribicola*.

## 3. Results and Analysis

### 3.1. Primer Screening

**Figure 1:**
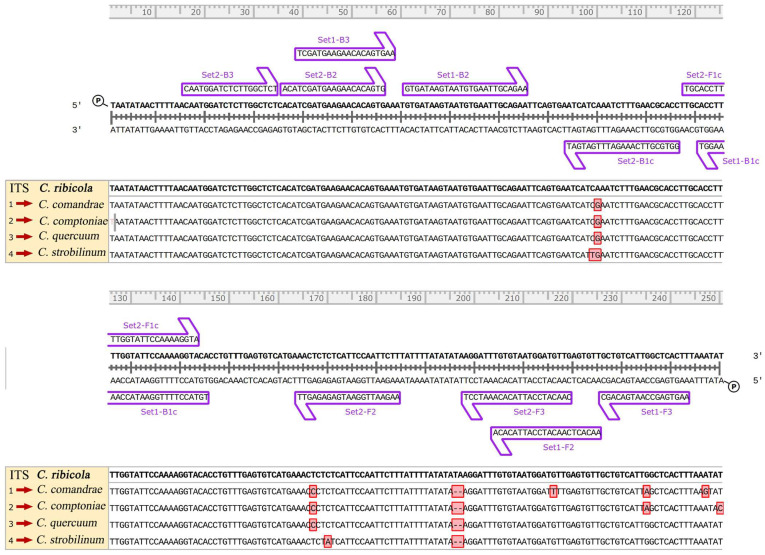
Sequence alignment of the target primers across the ITS regions of *C. ribicola* and four related *Cronartium* species.

Prior to in vitro validation, an in silico sequence alignment was performed to theoretically confirm the genomic uniqueness and binding specificity of the two candidate primer sets (Figure 1). Although the ITS regions exhibit high sequence homology among *Cronartium* species, alignment analysis revealed multiple species-specific single-nucleotide polymorphisms (SNPs) within the binding regions of both primer sets. Critically, distinct base mismatches (highlighted in red) were identified between *C. ribicola* and the four non-target species (*C. comandrae*, *C. comptoniae*, *C. quercuum*, and *C. strobilinum*). These diagnostic mismatches were strategically positioned within the key F2, B2, and Loop binding regions, disrupting the continuous base pairing required for Bst DNA polymerase extension and thereby theoretically preventing interspecific cross-reactivity.

**Figure 2:**
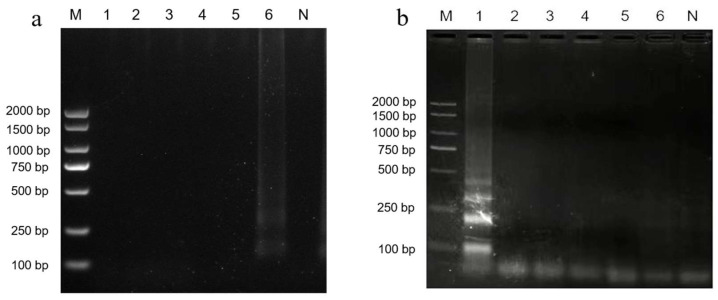
Selection and specificity screening of LAMP primers. (**a**) Electrophoresis screening results for Primer Set 1; (**b**) Electrophoresis screening results for Primer Set 2. M: DL2000 DNA Marker; 1: *Cronartium ribicola*; 2: *Melampsora larici-populina*; 3: *Gymnosporangiu-m asiaticum*; 4: *Erysiphe paeoniae*; 5: *Sawadaea tulasnei*; 6: *Flammulina velutipes*; N: Negative control (ddH_2_O).

Following in silico evaluation, the two candidate LAMP primer sets were assessed for amplification efficiency and analytical specificity. Agarose gel electrophoresis results are presented in Figure 2. Primer Set 1 (Figure 2a) failed to amplify *C. ribicola* genomic DNA (Lane 1) and exhibited non-specific amplification with a non-target fungal strain (Lane 6). In contrast, Primer Set 2 (Figure 2b) successfully and specifically amplified *C. ribicola* genomic DNA, producing characteristic ladder-like banding patterns (Lane 1), while the five remaining non-target fungal strains and the negative control (ddH_2_O) yielded no amplification bands. These results demonstrate that Primer Set 2 exhibited analytical specificity for *C. ribicola* within the defined test panel and was consequently selected for subsequent optimization and field evaluation.

### 3.2. Reaction System Optimization

**Figure 3:**
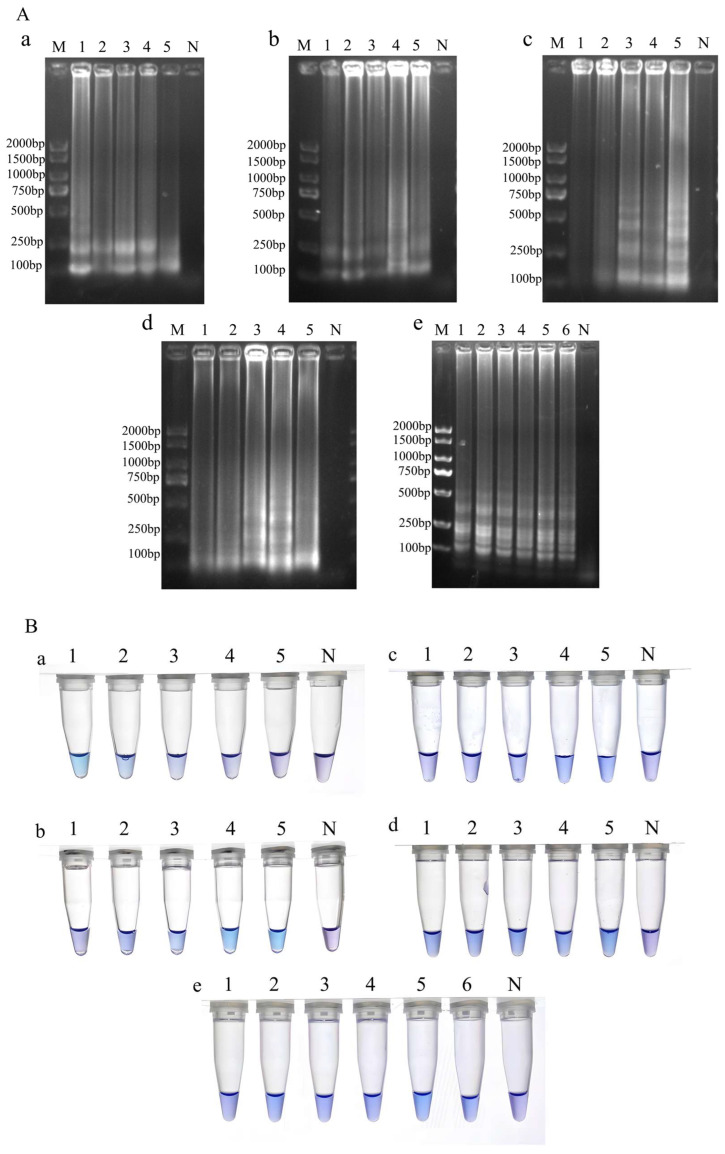
Optimization of the LAMP reaction system for *C. ribicola*. (**A**) Agarose gel electrophoresis results; (**B**) Colorimetric detection results using HNB dye. The sub-panels represent the single-factor optimization of: (**a**) Mg^2+^ concentration (Lanes/Tubes 1–5: 6.0, 8.0, 10.0, 12.0, and 14.0 mM; Lane 2 is the optimized 8.0 mM); (**b**) dNTP concentration (Lanes/Tubes 1–5: 0.8, 1.0, 1.2, 1.4, and 1.6 mM; Lane 2 is the optimized 1.0 mM); (**c**) Ratio of inner to outer primers (Lanes/Tubes 1–5: 1:1, 2:1, 4:1, 6:1, and 8:1; Lane 5 is the optimized 8:1); (**d**) Reaction time (Lanes/Tubes 1–5: 10, 20, 30, 40, and 50 min; Lane 4 is the optimized 40 min); (**e**) Amplification temperature (Lanes/Tubes 1–6: 61, 62, 63, 64, 65, and 66 °C; Lane 2 is the optimized 62 °C). M: DL2000 DNA Marker; N: Negative control (ddH_2_O).

Agarose gel electrophoresis results (Figure 3A) revealed the most intense and brightest characteristic ladder-like amplification bands in Lane 1 for Mg^2+^ (a), Lane 4 for dNTPs (b), Lane 5 for primer ratio (c), Lane 4 for reaction time (d), and Lane 2 for temperature (e). However, visual assessment using HNB dye (Figure 3B) imposed additional constraints on optimization. Specifically, at a Mg^2+^ concentration of 6.0 mM (Tube 1, Figure 3B(a)), the pre-reaction solution exhibited a bluish coloration that could compromise result interpretation and potentially introduce false-positive bias. Consequently, a Mg^2+^ concentration of 8.0 mM was selected. For dNTPs, a concentration of 1.0 mM was chosen as the minimum concentration producing distinct visual color differentiation. Integrating both electrophoretic and visual detection results, the optimized conditions for the 25 μL *C. ribicola* LAMP reaction were established as follows: Mg^2+^ concentration of 8.0 mM, dNTP concentration of 1.0 mM, inner-to-outer primer ratio of 8:1, amplification temperature of 62 °C, and reaction time of 40 min.

### 3.3. Sensitivity Detection

**Figure 4:**
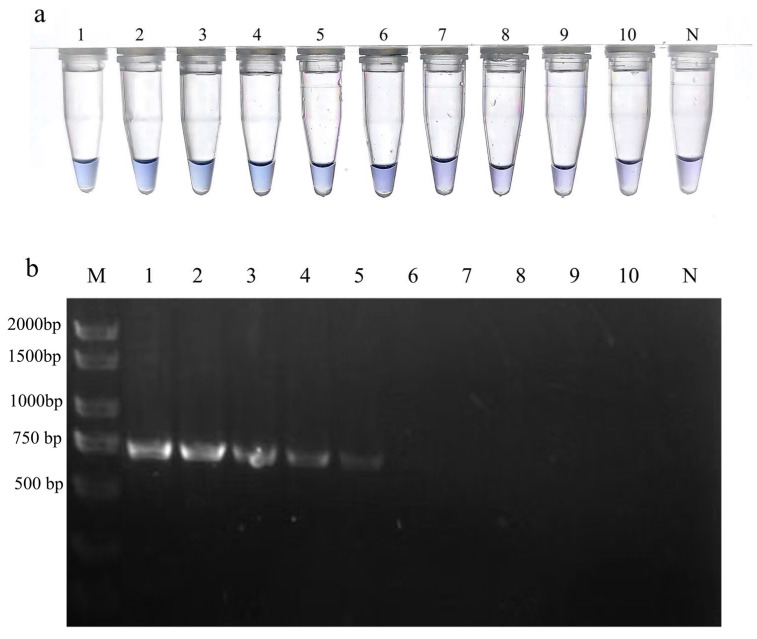
Sensitivity comparison between the LAMP assay and conventional PCR for detecting *C. ribicola*. (**a**) Visual sensitivity detection of the *C. ribicola* LAMP system using HNB dye; (**b**) Electrophoretic sensitivity detection of the conventional PCR system. Tubes/Lanes 1–10 correspond to genomic DNA concentrations of 46 ng/μL, 4.6 ng/μL, 460 pg/μL, 46 pg/μL, 4.6 pg/μL, 460 fg/μL, 46 fg/μL, 4.6 fg/μL, 460 ag/μL, and 46 ag/μL, respectively. N: Negative control (ddH_2_O).

Figure 4a illustrates the visual sensitivity of the *C. ribicola* LAMP assay, while Figure 4b presents the amplification results of conventional PCR. Tubes and lanes 1–10 represent a 10-fold serial dilution series of *C. ribicola* genomic DNA, with Lane/Tube N serving as the negative control (ddH_2_O). As shown in Figure 4a, the lowest DNA concentration producing a visually detectable positive color transition (purple to sky blue) corresponded to Tube 6 (460 ± 3.2 fg/μL), establishing the detection limit of the LAMP assay at 460 fg/μL. In contrast, conventional PCR (Figure 4b) yielded an extremely faint target amplification band at Lane 5 (4.6 pg/μL), setting the PCR detection limit at 4.6 ± 0.6 pg/μL. These comparative results demonstrate that under the tested conditions, the optimized LAMP reaction system achieved a detection limit of 460 fg/μL—a 10-fold improvement over conventional PCR—while eliminating the requirement for gel electrophoresis in result interpretation.

### 3.4. Sample Detection Results

This study systematically analyzed LAMP detection results of 211 *P. armandii* samples collected from four provinces in southwest China: Sichuan, Yunnan, Shaanxi, and Gansu. Samples were stratified into three temporal cohorts based on collection date: cohort A (May 2025), cohort B (April 2024), and cohort C (October 2023). Representative LAMP-HNB reaction tubes for all field samples are displayed in Figure S1. Detailed sample metadata and LAMP-HNB assay results, including color intensity classifications (0, 1, or 2), are comprehensively presented in Table S1.

Within the analyzed sample set, tissue type was strongly associated with detection outcome. Among samples collected from symptomatic *P. armandii* trees, the positive detection rate was significantly higher in bark tissues (68.97%, 20/29; 95% CI: 49.2–84.7%) than in needle tissues (18.75%, 3/16; 95% CI: 4.1–45.7%) (Table 5). A chi-square test revealed a significant effect of tissue type on the detection results (χ^2^ = 10.405, df = 1, *p* = 0.001). Fisher’s exact test further supported this conclusion (*p* = 0.002). Among samples from symptomatic trees, bark tissues showed a 50.2 percentage-point higher positive detection rate than needle tissues, corresponding to an approximately 3.68-fold higher detection rate. This discrepancy suggests a tissue-specific distribution of the blister rust pathogen within the host, exhibiting a tendency to accumulate more readily in the trunk and branch cortex, potentially shifting from needles to the bark for long-term colonization. Therefore, bark represents a more reliable tissue than needles for pathogen detection in symptomatic plants.

Notably, no positive results were found among the 82 asymptomatic needle samples (0.00%, 95% CI: 0.0–4.4%), whereas 3 cases (3.75%, 95% CI: 0.8–10.6%) were detected in the 80 asymptomatic bark samples. These findings suggest that bark is superior to needles for diagnosis in both symptomatic and asymptomatic trees, positioning bark as the preferred tissue for early pathogen screening prior to symptom manifestation. However, since DNA-based assays cannot distinguish between viable pathogens, residual nucleic acids, and inactive surface spores, positive results in asymptomatic samples indicate the presence of *C. ribicola* DNA but cannot definitively confirm latent infection status. Additionally, the sky-blue coloration in positive reactions from field samples was consistently lighter than that observed in pure pathogen DNA controls (Figure S1a). As expected, both CR1 and CR2 controls demonstrated identical amplification dynamics across all validation assays. The reduced color intensity in plant extracts likely reflects lower initial DNA template concentrations or the presence of inhibitory substances within the tissue matrix. This underscores the importance of employing appropriate reference controls when interpreting visual results in field diagnostic applications.

**Figure 5:**
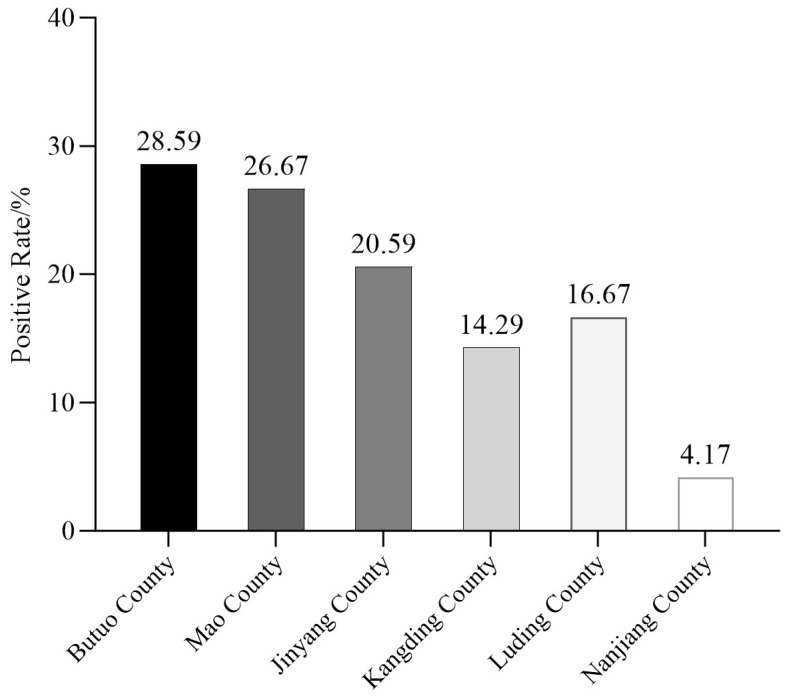
Distribution of positive detection rates for *Cronartium ribicola* by LAMP assay in *P. armandii* forest across different counties.

Spatially, positive detections showed a site-specific pattern in the surveyed regions. Positive samples were mainly recorded at several sampling sites in southwestern Sichuan Province, most of which were located above 2000 m a.s.l., including Butuo County (28.59%), Mao County (26.67%), Jinyang County (20.59%), Luding County (16.67%), and Kangding County (14.29%) (Figure 5). In contrast, no positive reactions were observed in the tested samples from Qiaojia County in Yunnan Province; Feng County, Liuba County, and Huayin County in Shaanxi Province; or Liangdang County (Xiaolongshan) in Gansu Province. These results suggest that positive detections were associated with specific sampled locations, several of which were situated above 2000 m a.s.l.

## 4. Discussion

This study successfully established a visual LAMP-HNB detection system for *Cronartium ribicola* with demonstrated analytical specificity, high sensitivity, and practical applicability. The specificity of Primer Set 2 was dually validated through in silico sequence alignment and in vitro experimental confirmation. Alignment analysis identified multiple species-specific SNPs within primer binding regions that effectively prevent cross-reactivity with related *Cronartium* species. Furthermore, the absence of cross-amplification with common sympatric forest fungi ensures robust detection reliability in complex ecological environments, thereby establishing a reliable foundation for accurate disease identification in field settings. Systematic optimization of reaction parameters yielded the following conditions: 8 mmol·L^−1^ Mg^2+^, 1 mmol·L^−1^ dNTPs, inner-to-outer primer ratio of 8:1 at 10 μmol·L^−1^, and reaction time of 40 min at 62 °C. This optimized protocol not only ensures detection stability and improves detection efficiency but also demonstrates suitability for large-scale sample screening applications.

Regarding analytical sensitivity, the system achieved a detection limit of 460 fg·μL^−1^ for *C. ribicola* genomic DNA, enabling reliable identification of early infections and samples with low pathogen loads. Under the tested conditions, this detection limit represents a 10-fold improvement over conventional PCR, providing robust technical support for early disease warning and field-based detection following DNA extraction. The incorporation of hydroxynaphthol blue (HNB) as a colorimetric indicator further enhances the system’s practical utility, eliminating the need for post-amplification processing and enabling rapid visual result interpretation in field settings [23]. The HNB-based colorimetric readout, displaying a sky-blue coloration for positive reactions and purple coloration for negative reactions, permits visual result assessment when interpreted alongside appropriate positive and negative controls. This approach reduces dependence on specialized thermal cycling and electrophoresis equipment following DNA extraction while mitigating the risk of aerosol contamination associated with post-amplification tube manipulation. This makes the assay suitable for application in primary-level forestry stations and field-oriented monitoring scenarios where basic DNA extraction facilities are available. Compared with the previously reported field-ready LAMP assay for *C. ribicola* based on North American WPBR samples [20], the present study provides complementary validation and application data for Chinese *P. armandii* forest systems. Notably, this study optimized an HNB-based visual LAMP assay, validated its analytical specificity against related *Cronartium* species and common sympatric fungi, and evaluated its performance on 211 field-collected *P. armandii* samples from high-altitude regions. These findings extend the practical application of LAMP-based *C. ribicola* detection to Chinese white pine forest ecosystems and establish baseline epidemiological data for WPBR surveillance and management in China.

Furthermore, the significantly higher detection rate in symptomatic bark (68.97%) compared to needles (18.75%) suggests that the pathogen migrates and accumulates in the bark [24], making it a more reliable diagnostic target. Notably, the exclusive detection of *C. ribicola* DNA in asymptomatic bark samples (3.75%) compared to the absence of detections in asymptomatic needles (0%) underscores the critical importance of bark tissue for pre-symptomatic pathogen screening. However, since DNA-based assays cannot differentiate between active infections and non-viable residual nucleic acids, complementary histological or RNA-based analyses are necessary to definitively confirm active latent infections.

Additionally, the reduced color intensity observed in certain positive field samples suggests potential amplification inhibition by secondary metabolites (e.g., polyphenols and resins) prevalent in coniferous tissues. The absence of an internal amplification control and extraction control represents a current limitation of the assay. Future optimization should consider the incorporation of spiked controls, extraction controls, internal amplification controls, and template dilution assays to systematically evaluate and mitigate inhibition in resinous pine tissues.

Given its high sensitivity and simplified visual readout, the LAMP-HNB technology demonstrates potential for rapid pathogen screening and epidemic early-warning systems following DNA extraction. This capability has immediate applications in two key areas. Within seedling quarantine and certification programs, the assay can be deployed to assess infection rates in nurseries prior to seedling distribution, thereby preventing disease establishment at the source. This rapid quarantine screening effectively interrupts human-mediated disease transmission pathways across geographic regions. Similar LAMP-based diagnostic systems have been successfully implemented for quarantine-regulated plant pathogens (e.g., *Curtobacterium flaccumfaciens*), demonstrating effectiveness in both port inspections and field surveillance [25]. Additionally, forestry management agencies can establish fixed-point monitoring networks utilizing this technology to systematically assess pathogen distribution and population dynamics in key forest ecosystems. This approach provides robust epidemiological data to support prediction of outbreak trends and evaluation of management intervention efficacy, including alternate host removal and stand density modification.

Field detection results revealed that *C. ribicola* DNA was predominantly identified at discrete sampling locations in southwestern China, predominantly distributed at high elevations. Positive detections were rare in the surveyed regions below 2000 m, except for asymptomatic samples from Nanjiang County in the Bashan Mountains at approximately 1800 m [26]. This spatial pattern suggests that positive detections in the surveyed regions are associated with specific sampling locations and their corresponding local environmental conditions, whereas the relationship between elevation and disease occurrence requires further systematic investigation through expanded sampling efforts. Historically, *P. armandii* is distributed across elevations from 1000 to 3500 m [27], and WPBR was documented at elevations from 1500 to 1800 m in the Qinling–Bashan Mountains approximately two decades ago [9]. However, it is difficult to be sampled recent years even at the same place with multiple-aged *P. armandii*. Some researchers attribute this apparent decline to rising global temperatures and alterations in host defense pathways, proposing that elevated temperatures enhance effector-triggered immunity (ETI) in *P. armandii*, thereby enabling infected trees to remain asymptomatic despite *C. ribicola* infection [28]. It has been hypothesized that WPBR prevalence may decline under warmer and drier climatic conditions, given the pathogen’s ecological requirement for cool, moist environments to facilitate successful infection [29]. A comprehensive analysis integrating multiple environmental variables—including stream density, topography, hardiness zones, precipitation, air temperature, vapor pressure deficit, and relative humidity—demonstrated that WPBR risk increases with increasing moisture availability and declines with rising air temperature [10]. Studies conducted in the Rocky Mountains in the USA have found that trees inhabiting more arid environments are less susceptible to infection by *C. ribicola* [30], thereby supporting this hypothesis. However, in areas experiencing increasing aridity, trees already infected with the fungus are more likely to exhibit severe symptoms and mortality. In contrast, it was proposed that increased aridity contributed to a shift in the prevalence of WPBR in the Southern Sierra Nevada Mountains [29]. Furthermore, rising temperatures may intensify WPBR pressure in moist habitats by extending the growing season, thereby prolonging the infectious period and facilitating extensive canker development. These findings underscore the complexity of predicting WPBR risk under changing climatic conditions and provide a foundation for tailoring landscape restoration strategies to local climatic characteristics.

## 5. Conclusions

This study successfully established a visual LAMP-based detection system for *Cronartium ribicola*. Through systematic primer optimization and reaction condition refinement, the following parameters were determined to be optimal: Mg^2+^ concentration of 8 mmol·L^−1^, dNTP concentration of 1 mmol·L^−1^, inner-to-outer primer ratio of 8:1, and amplification time of 40 min at 62 °C. The assay achieved a detection limit of 460 fg·μL^−1^, representing a 10-fold improvement over conventional PCR. Integration of the hydroxynaphthol blue (HNB) chromogenic indicator enables rapid visual readout without post-amplification processing: positive reactions display sky-blue coloration, while negative reactions remain purple, with interpretation guided by appropriate positive and negative controls. Field application of this system on 211 *P. armandii* samples from southwestern China revealed that positive *C. ribicola* detections were predominantly clustered at discrete sampling locations, with a notable association with elevated elevations. By combining high sensitivity with operational simplicity and visual interpretability, this LAMP-HNB assay addresses a gap in molecular diagnostic capabilities for *C. ribicola* and provides baseline data for monitoring WPBR distribution patterns and epidemiological dynamics under changing climatic conditions.

## Figures and Tables

**Table 1:** Sampling information of *Pinus armandii* materials (“-”refer to no sample).

Locus	Host	Altitude Range (m)	Diseased Number			Asymptomatic Number		Total	Aeciospore
			Bark	Needle	Root	Bark	Needle		
Butuo	*P. armandii*	2092~2919	15	7	2	16	9	49	Yes
Mao	*P. armandii*	2030~2061	4	1	-	5	5	15	Yes
Jinyang	*P. armandii*	2562~3103	7	8	2	8	9	34	Yes
Kangding	*P. armandii*	2692~2702	2	2	-	2	1	7	Yes
Luding	*P. armandii*	2532~2570	-	-	-	3	3	6	-
Nanjiang	*P. armandii*	1208~1952	-	-	-	12	12	24	-
Zhaojue	*P. armandii*	2185~2780	-	-	-	9	8	17	-
Meigu	*P. armandii*	2685	-	-	-	1	1	2	-
Danba	*P. armandii*	1880~2636	-	-	-	5	5	10	-
Jiangyou	*P. armandii*	1586~1642	-	-	-	4	4	8	-
Liuba	*P. armandii*	1719~1862	-	-	-	4	4	8	-
Huidong	*P. armandii*	2605	-	-	-	3	3	6	-
Zhouzhi	*P. armandii*	1878	-	-	-	2	2	4	-
Huayin	*P. armandii*	1614~2083	-	-	-	2	2	4	-
Feng	*P. armandii*	1736				-	1	1	-
Liangdang	*P. armandii*	1618~1932	-	-	-	2	10	12	-
Qiaojia	*P. armandii*	1980	-	-	-	2	2	4	-
Benxi	*P. koraiensis*	443	-	-	-	-	-	0	Yes

**Table 2:** Primer sets for LAMP detection.

Primer Set	Primer Name	Primer Sequences
Primer set 1	FIP	GTGTCATGAAACTCTCTCATTCCAAAACACTCAACATCCATTACACA
	BIP	TGTACCTTTTGGAATACCAAAAGGTGTGATAAGTAATGTGAATTGCAGAA
	F3	AAGTGAGCCAATGACAGC
	B3	TCGATGAAGAACACAGTGAA
Primer set 2	FIP	TGCACCTTTTGGTATTCCAAAAGGTAAAGAATTGGAATGAGAGAGTT
	BIP	GGTGCGTTCAAAGATTTGATGATTACATCGATGAAGAACACAGTG
	F3	CAACATCCATTACACAAATCCT
	B3	CAATGGATCTCTTGGCTCT

**Table 3:** Initial reaction system.

Components and Concentrations	Volume/μL
10 × Buffer	2.5
Mg^2+^ (100 mmol·L^−1^)	1.5
dNTPs (10 mmol·L^−1^ each)	3.5
FIP (10 μmol·L^−1^)	4
BIP (10 μmol·L^−1^)	4
F3 (10 μmol·L^−1^)	0.5
B3 (10 μmol·L^−1^)	0.5
template	1.5
Bst DNA polymerase (8000 U·mL^−1^)	0.5
ddH_2_O	Up to 25

**Table 4:** Gradient optimization of LAMP reaction components and conditions.

Factor	Gradient
Mg^2+^ concentration (mmol·L^−1^)	6, 8, 10, 12, 14
dNTP concentration (mmol·L^−1^)	0.8, 1, 1.2, 1.4, 1.6
Ratio of inner to outer primers	1:1, 2:1, 4:1, 6:1, 8:1
Temperature (℃)	61, 62, 63, 64, 65, 66
Time (min)	10, 20, 30, 40, 50

**Table 5:** Positive detection rates among asymptomatic and diseased *P. armandii* tissues.

Health Status	Tissue Type	Number of Samples	Number of Positives	Positive Rate (%)	95% CI
Asymptomatic	Needles	82	0	0	0.0–4.4%
Asymptomatic	Bark	80	3	3.75	0.8–10.6%
Diseased	Needles	16	3	18.75	4.1–45.7%
Diseased	Bark	29	20	68.97	49.2–84.7%
Diseased	Roots	4	2	50.00	6.8–93.2%
Total		211	28	13.27	9.0–18.6%

## Data Availability

The original contributions presented in the study are included in the article; further inquiries can be directed to the corresponding author.

## Supplementary Materials

The following supporting information can be downloaded at: https://www.mdpi.com/article/10.3390/jof12060409/s1, Figure S1: Complete LAMP-HNB reaction-tube panel for field-collected *Pinus armandii* samples; Table S1: Detailed metadata of field samples and HNB reaction.
