# Supplementary material for: Establishment of a Visual LAMP Technology and Detection of Cronartium ribicola Infecting Chinese White Pine in Southwestern China
Source: J Fungi (Basel). 2026 Jun 4;12(6):409. doi: 10.3390/jof12060409 (PMC13301191; doi:10.3390/jof12060409)
Supplement: Supplementary file 1 [file jof-12-00409-s001.zip › Figure S1.pdf]

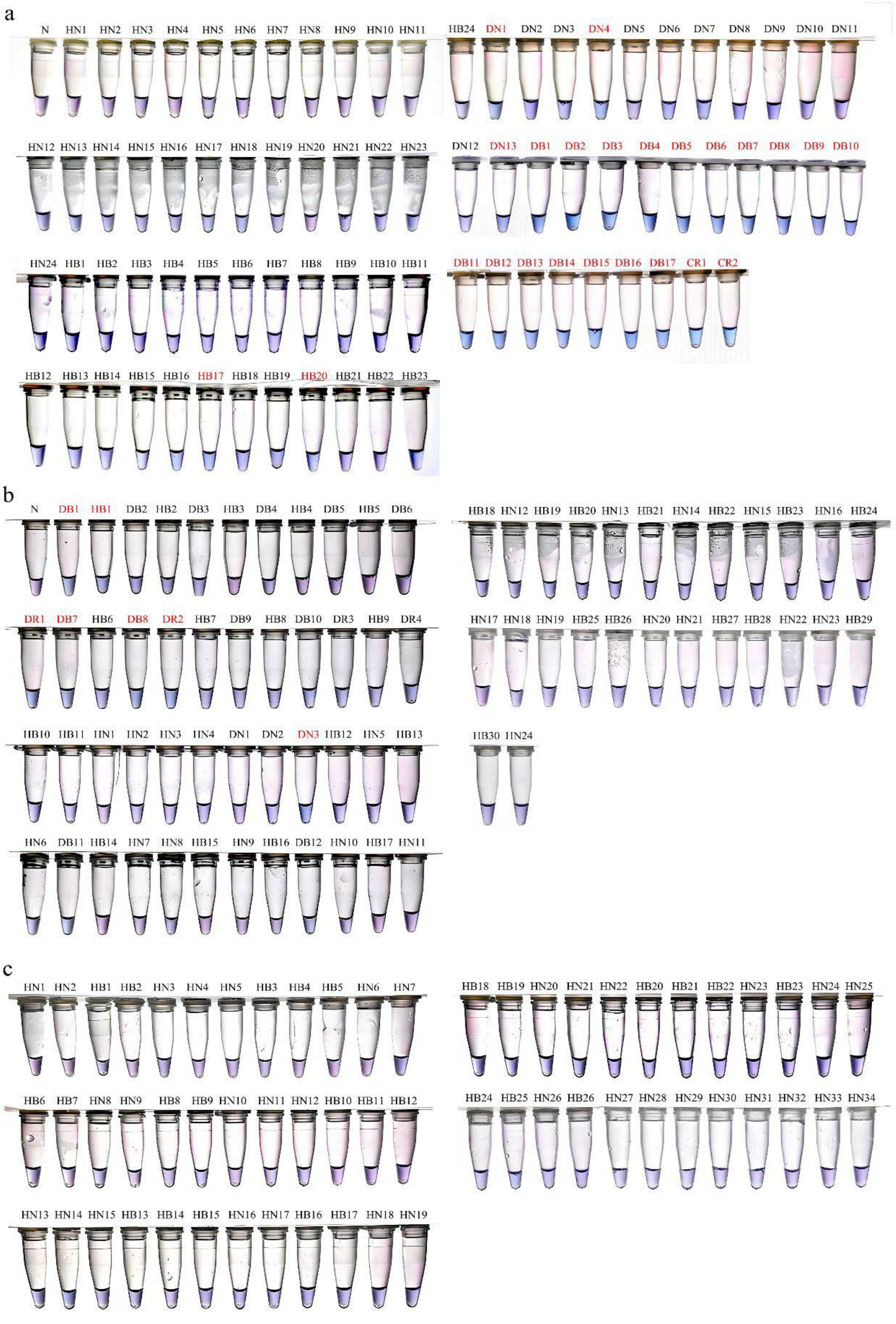

Figure S1. Complete LAMP-HNB reaction-tube panel for field-collected *Pinus armandii* samples.

The samples were divided into three batches based on collection time: (a) May 2025; (b) April 2024; and (c) October 2023. A total of 211 samples from Sichuan, Yunnan, Shaanxi, and Gansu provinces were analyzed. Each tube represents one reaction. HB: asymptomatic bark samples; HN: asymptomatic needle samples; DB: diseased bark samples; DN: diseased needle samples; DR: diseased root samples. Red labels indicate positive reactions, and black labels indicate negative reactions, as interpreted with appropriate positive and negative controls.
