# Supplementary material for: Establishment of a Visual LAMP Technology and Detection of Cronartium ribicola Infecting Chinese White Pine in Southwestern China
Source: J Fungi (Basel). 2026 Jun 4;12(6):409. doi: 10.3390/jof12060409 (PMC13301191; doi:10.3390/jof12060409)
Supplement: Supplementary file 1 [file jof-12-00409-s001.zip › Table S1. Detailed metadata of field samples and HNB.pdf]

Table S1. Detailed metadata of field samples and HNB-LAMP assay results

Sample ID	Collection Date	Province	County	Altitude	Tissue Type	Symptom Status	LAMP Result	Color Intensity
A_HN1	202505	Sichuan	Mao	2030	Needle	Asymptomatic	Negative	0
A_HN2	202505	Sichuan	Butuo	2784	Needle	Asymptomatic	Negative	0
A_HN3	202505	Sichuan	Butuo	2784	Needle	Asymptomatic	Negative	0
A_HN4	202505	Sichuan	Butuo	2784	Needle	Asymptomatic	Negative	0
A_HN5	202505	Sichuan	Butuo	2548	Needle	Asymptomatic	Negative	0
A_HN6	202505	Sichuan	Butuo	2113	Needle	Asymptomatic	Negative	0
A_HN7	202505	Sichuan	Butuo	2092	Needle	Asymptomatic	Negative	0
A_HN8	202505	Sichuan	Jinyang	2715	Needle	Asymptomatic	Negative	0
A_HN9	202505	Sichuan	Jinyang	2645	Needle	Asymptomatic	Negative	0
A_HN10	202505	Sichuan	Jinyang	2658	Needle	Asymptomatic	Negative	0
A_HN11	202505	Sichuan	Jinyang	2653	Needle	Asymptomatic	Negative	0
A_HN12	202505	Sichuan	Zhaojue	2185	Needle	Asymptomatic	Negative	0
A_HN13	202505	Sichuan	Zhaojue	2357	Needle	Asymptomatic	Negative	0
A_HN14	202505	Sichuan	Zhaojue	2780	Needle	Asymptomatic	Negative	0
A_HN15	202505	Sichuan	Meigu	2685	Needle	Asymptomatic	Negative	0
A_HN16	202505	Sichuan	Kangding	2692	Needle	Asymptomatic	Negative	0
A_HN17	202505	Sichuan	Luding	2532	Needle	Asymptomatic	Negative	0
A_HN18	202505	Sichuan	Danba	2544	Needle	Asymptomatic	Negative	0
A_HN19	202505	Sichuan	Nanjiang	1208	Needle	Asymptomatic	Negative	0
A_HN20	202505	Sichuan	Nanjiang	1208	Needle	Asymptomatic	Negative	0
A_HN21	202505	Sichuan	Nanjiang	1208	Needle	Asymptomatic	Negative	0
A_HN22	202505	Sichuan	Nanjiang	1208	Needle	Asymptomatic	Negative	0
A_HN23	202505	Sichuan	Nanjiang	1208	Needle	Asymptomatic	Negative	0
A_HN24	202505	Sichuan	Nanjiang	1208	Needle	Asymptomatic	Negative	0
A_HB1	202505	Sichuan	Mao	2030	Bark	Asymptomatic	Negative	0
A_HB2	202505	Sichuan	Butuo	2784	Bark	Asymptomatic	Negative	0
A_HB3	202505	Sichuan	Butuo	2784	Bark	Asymptomatic	Negative	0
A_HB4	202505	Sichuan	Butuo	2784	Bark	Asymptomatic	Negative	0
A_HB5	202505	Sichuan	Butuo	2548	Bark	Asymptomatic	Negative	0
A_HB6	202505	Sichuan	Butuo	2113	Bark	Asymptomatic	Negative	0
A_HB7	202505	Sichuan	Butuo	2092	Bark	Asymptomatic	Negative	0
A_HB8	202505	Sichuan	Jinyang	2715	Bark	Asymptomatic	Negative	0
A_HB9	202505	Sichuan	Jinyang	2645	Bark	Asymptomatic	Negative	0
A_HB10	202505	Sichuan	Jinyang	2658	Bark	Asymptomatic	Negative	0
A_HB11	202505	Sichuan	Jinyang	2653	Bark	Asymptomatic	Negative	0
A_HB12	202505	Sichuan	Zhaojue	2185	Bark	Asymptomatic	Negative	0
A_HB13	202505	Sichuan	Zhaojue	2357	Bark	Asymptomatic	Negative	0
A_HB14	202505	Sichuan	Zhaojue	2780	Bark	Asymptomatic	Negative	0
A_HB15	202505	Sichuan	Meigu	2685	Bark	Asymptomatic	Negative	0
A_HB16	202505	Sichuan	Kangding	2692	Bark	Asymptomatic	Negative	0

A_HB17	202505	Sichuan	Luding	2532	Bark	Asymptomatic	Positive	1
A_HB18	202505	Sichuan	Danba	2544	Bark	Asymptomatic	Negative	0
A_HB19	202505	Sichuan	Nanjiang	1208	Bark	Asymptomatic	Negative	0
A_HB20	202505	Sichuan	Nanjiang	1208	Bark	Asymptomatic	Positive	1
A_HB21	202505	Sichuan	Nanjiang	1208	Bark	Asymptomatic	Negative	0
A_HB22	202505	Sichuan	Nanjiang	1208	Bark	Asymptomatic	Negative	0
A_HB23	202505	Sichuan	Nanjiang	1208	Bark	Asymptomatic	Negative	0
A_HB24	202505	Sichuan	Nanjiang	1208	Bark	Asymptomatic	Negative	0
A_DN1	202505	Sichuan	Mao	2030	Needle	Symptomatic	Positive	2
A_DN2	202505	Sichuan	Butuo	2784	Needle	Symptomatic	Negative	0
A_DN3	202505	Sichuan	Butuo	2784	Needle	Symptomatic	Negative	0
A_DN4	202505	Sichuan	Butuo	2784	Needle	Symptomatic	Positive	2
A_DN5	202505	Sichuan	Butuo	2548	Needle	Symptomatic	Negative	0
A_DN6	202505	Sichuan	Butuo	2092	Needle	Symptomatic	Negative	0
A_DN7	202505	Sichuan	Butuo	2092	Needle	Symptomatic	Negative	0
A_DN8	202505	Sichuan	Butuo	2092	Needle	Symptomatic	Negative	0
A_DN9	202505	Sichuan	Jinyang	2715	Needle	Symptomatic	Negative	0
A_DN10	202505	Sichuan	Jinyang	2645	Needle	Symptomatic	Negative	0
A_DN11	202505	Sichuan	Jinyang	2653	Needle	Symptomatic	Negative	0
A_DN12	202505	Sichuan	Jinyang	2653	Needle	Symptomatic	Negative	0
A_DN13	202505	Sichuan	Kangding	2692	Needle	Symptomatic	Positive	2
A_DB1	202505	Sichuan	Mao	2030	Bark	Symptomatic	Positive	2
A_DB2	202505	Sichuan	Mao	2030	Bark	Symptomatic	Positive	2
A_DB3	202505	Sichuan	Mao	2030	Bark	Symptomatic	Positive	2
A_DB4	202505	Sichuan	Butuo	2784	Bark	Symptomatic	Positive	2
A_DB5	202505	Sichuan	Butuo	2784	Bark	Symptomatic	Positive	2
A_DB6	202505	Sichuan	Butuo	2784	Bark	Symptomatic	Positive	2
A_DB7	202505	Sichuan	Butuo	2548	Bark	Symptomatic	Positive	2
A_DB8	202505	Sichuan	Butuo	2092	Bark	Symptomatic	Positive	2
A_DB9	202505	Sichuan	Butuo	2092	Bark	Symptomatic	Positive	2
A_DB10	202505	Sichuan	Butuo	2092	Bark	Symptomatic	Positive	2
A_DB11	202505	Sichuan	Jinyang	2715	Bark	Symptomatic	Positive	2
A_DB12	202505	Sichuan	Jinyang	2715	Bark	Symptomatic	Positive	2
A_DB13	202505	Sichuan	Jinyang	2699	Bark	Symptomatic	Positive	2
A_DB14	202505	Sichuan	Jinyang	2645	Bark	Symptomatic	Positive	2
A_DB15	202505	Sichuan	Jinyang	2653	Bark	Symptomatic	Positive	2
A_DB16	202505	Sichuan	Jinyang	2653	Bark	Symptomatic	Positive	2
A_DB17	202505	Sichuan	Kangding	2692	Bark	Symptomatic	Positive	2
B_DB1	202404	Sichuan	Butuo	2560	Bark	Symptomatic	Positive	1
B_HB1	202404	Sichuan	Butuo	2919	Bark	Asymptomatic	Positive	1
B_DB2	202404	Sichuan	Butuo	2919	Bark	Symptomatic	Negative	0
B_HB2	202404	Sichuan	Butuo	2886	Bark	Asymptomatic	Negative	0
B_DB3	202404	Sichuan	Butuo	2886	Bark	Symptomatic	Negative	0
B_HB3	202404	Sichuan	Butuo	2895	Bark	Asymptomatic	Negative	0

B_DB4	202404	Sichuan	Butuo	2895	Bark	Symptomatic	Negative	0
B_HB4	202404	Sichuan	Butuo	2859	Bark	Asymptomatic	Negative	0
B_DB5	202404	Sichuan	Butuo	2859	Bark	Symptomatic	Negative	0
B_HB5	202404	Sichuan	Butuo	2838	Bark	Asymptomatic	Negative	0
B_DB6	202404	Sichuan	Butuo	2838	Bark	Symptomatic	Negative	0
B_DR1	202404	Sichuan	Butuo	2838	Root	Symptomatic	Positive	1
B_DB7	202404	Sichuan	Butuo	2625	Bark	Symptomatic	Positive	1
B_HB6	202404	Sichuan	Butuo	2625	Bark	Asymptomatic	Negative	0
B_DB8	202404	Sichuan	Butuo	2625	Bark	Symptomatic	Positive	1
B_DR2	202404	Sichuan	Butuo	2625	Root	Symptomatic	Positive	1
B_HB7	202404	Sichuan	Jinyang	2782	Bark	Asymptomatic	Negative	0
B_DB9	202404	Sichuan	Jinyang	2782	Bark	Symptomatic	Negative	0
B_HB8	202404	Sichuan	Jinyang	2996	Bark	Asymptomatic	Negative	0
B_DB10	202404	Sichuan	Jinyang	3103	Bark	Symptomatic	Negative	0
B_DR3	202404	Sichuan	Jinyang	3103	Root	Symptomatic	Negative	0
B_HB9	202404	Sichuan	Zhaojue	2512	Bark	Asymptomatic	Negative	0
B_DR4	202404	Sichuan	Jinyang	2996	Root	Symptomatic	Negative	0
B_HB10	202404	Sichuan	Zhaojue	2512	Bark	Asymptomatic	Negative	0
B_HB11	202404	Sichuan	Butuo	2632	Bark	Asymptomatic	Negative	0
B_HN1	202404	Sichuan	Jinyang	2996	Needle	Asymptomatic	Negative	0
B_HN2	202404	Sichuan	Jinyang	2996	Needle	Asymptomatic	Negative	0
B_HN3	202404	Sichuan	Jinyang	2782	Needle	Asymptomatic	Negative	0
B_HN4	202404	Sichuan	Zhaojue	2512	Needle	Asymptomatic	Negative	0
B_DN1	202404	Sichuan	Jinyang	3103	Needle	Symptomatic	Negative	0
B_DN2	202404	Sichuan	Jinyang	3103	Needle	Symptomatic	Negative	0
B_DN3	202404	Sichuan	Jinyang	2782	Needle	Symptomatic	Positive	1
B_HB12	202404	Sichuan	Luding	2570	Bark	Asymptomatic	Negative	0
B_HN5	202404	Sichuan	Luding	2570	Needle	Asymptomatic	Negative	0
B_HB13	202404	Sichuan	Kangding	2707	Bark	Asymptomatic	Negative	0
B_HN6	202404	Sichuan	Kangding	2707	Needle	Asymptomatic	Negative	0
B_DB11	202404	Sichuan	Kangding	2707	Bark	Symptomatic	Negative	0
B_HB14	202404	Sichuan	Danba	2636	Bark	Asymptomatic	Negative	0
B_HN7	202404	Sichuan	Danba	2636	Needle	Asymptomatic	Negative	0
B_HN8	202404	Sichuan	Danba	2636	Needle	Asymptomatic	Negative	0
B_HB15	202404	Sichuan	Danba	2636	Bark	Asymptomatic	Negative	0
B_HN9	202404	Sichuan	Mao	2061	Needle	Asymptomatic	Negative	0
B_HB16	202404	Sichuan	Mao	2061	Bark	Asymptomatic	Negative	0
B_DB12	202404	Sichuan	Mao	2061	Bark	Symptomatic	Negative	0
B_HN10	202404	Sichuan	Mao	2045	Needle	Asymptomatic	Negative	0
B_HB17	202404	Sichuan	Mao	2045	Bark	Asymptomatic	Negative	0
B_HN11	202404	Sichuan	Jiangyou	1594	Needle	Asymptomatic	Negative	0
B_HB18	202404	Sichuan	Jiangyou	1594	Bark	Asymptomatic	Negative	0
B_HN12	202404	Sichuan	Jiangyou	1642	Needle	Asymptomatic	Negative	0
B_HB19	202404	Sichuan	Jiangyou	1642	Bark	Asymptomatic	Negative	0

B_HB20	202404	Sichuan	Nanjiang	1952	Bark	Asymptomatic	Negative	0
B_HN13	202404	Sichuan	Nanjiang	1952	Needle	Asymptomatic	Negative	0
B_HB21	202404	Sichuan	Nanjiang	1591	Bark	Asymptomatic	Negative	0
B_HN14	202404	Sichuan	Nanjiang	1591	Needle	Asymptomatic	Negative	0
B_HB22	202404	Sichuan	Nanjiang	1842	Bark	Asymptomatic	Negative	0
B_HN15	202404	Sichuan	Nanjiang	1842	Needle	Asymptomatic	Negative	0
B_HB23	202404	Shaanxi	Liuba	1862	Bark	Asymptomatic	Negative	0
B_HN16	202404	Shaanxi	Liuba	1862	Needle	Asymptomatic	Negative	0
B_HB24	202404	Shaanxi	Liuba	1809	Bark	Asymptomatic	Negative	0
B_HN17	202404	Shaanxi	Liuba	1809	Needle	Asymptomatic	Negative	0
B_HN18	202404	Gansu	Liangdang	1932	Needle	Asymptomatic	Negative	0
B_HN19	202404	Gansu	Liangdang	1932	Needle	Asymptomatic	Negative	0
B_HB25	202404	Gansu	Liangdang	1932	Bark	Asymptomatic	Negative	0
B_HB26	202404	Gansu	Liangdang	1932	Bark	Asymptomatic	Negative	0
B_HN20	202404	Shaanxi	Zhouzhi	1878	Needle	Asymptomatic	Negative	0
B_HN21	202404	Shaanxi	Zhouzhi	1878	Needle	Asymptomatic	Negative	0
B_HB27	202404	Shaanxi	Zhouzhi	1878	Bark	Asymptomatic	Negative	0
B_HB28	202404	Shaanxi	Zhouzhi	1878	Bark	Asymptomatic	Negative	0
B_HN22	202404	Shaanxi	Huayin	1614	Needle	Asymptomatic	Negative	0
B_HN23	202404	Shaanxi	Huayin	1614	Needle	Asymptomatic	Negative	0
B_HB29	202404	Shaanxi	Huayin	2083	Bark	Asymptomatic	Negative	0
B_HB30	202404	Shaanxi	Huayin	2083	Bark	Asymptomatic	Negative	0
B_HN24	202404	Shaanxi	Feng	1736	Needle	Asymptomatic	Negative	0
C_HN1	202310	Yunnan	Qiaojia	1980	Needle	Asymptomatic	Negative	0
C_HN2	202310	Yunnan	Qiaojia	1980	Needle	Asymptomatic	Negative	0
C_HB1	202310	Yunnan	Qiaojia	1980	Bark	Asymptomatic	Negative	0
C_HB2	202310	Yunnan	Qiaojia	1980	Bark	Asymptomatic	Negative	0
C_HN3	202310	Sichuan	Butuo	2648	Needle	Asymptomatic	Negative	0
C_HN4	202310	Sichuan	Butuo	2648	Needle	Asymptomatic	Negative	0
C_HN5	202310	Sichuan	Butuo	2693	Needle	Asymptomatic	Negative	0
C_HB3	202310	Sichuan	Butuo	2648	Bark	Asymptomatic	Negative	0
C_HB4	202310	Sichuan	Butuo	2648	Bark	Asymptomatic	Negative	0
C_HB5	202310	Sichuan	Butuo	2693	Bark	Asymptomatic	Negative	0
C_HN6	202310	Sichuan	Jinyang	2706	Needle	Asymptomatic	Negative	0
C_HN7	202310	Sichuan	Jinyang	2562	Needle	Asymptomatic	Negative	0
C_HB6	202310	Sichuan	Jinyang	2706	Bark	Asymptomatic	Negative	0
C_HB7	202310	Sichuan	Jinyang	2562	Bark	Asymptomatic	Negative	0
C_HN8	202310	Sichuan	Jiangyou	1586	Needle	Asymptomatic	Negative	0
C_HN9	202310	Sichuan	Jiangyou	1586	Needle	Asymptomatic	Negative	0
C_HB8	202310	Sichuan	Jiangyou	1586	Bark	Asymptomatic	Negative	0
C_HB9	202310	Sichuan	Jiangyou	1586	Bark	Asymptomatic	Negative	0
C_HN10	202310	Sichuan	Nanjiang	1531	Needle	Asymptomatic	Negative	0
C_HN11	202310	Sichuan	Nanjiang	1531	Needle	Asymptomatic	Negative	0
C_HN12	202310	Sichuan	Nanjiang	1531	Needle	Asymptomatic	Negative	0

C_HB10	202310	Sichuan	Nanjiang	1531	Bark	Asymptomatic	Negative	0
C_HB11	202310	Sichuan	Nanjiang	1531	Bark	Asymptomatic	Negative	0
C_HB12	202310	Sichuan	Nanjiang	1531	Bark	Asymptomatic	Negative	0
C_HN13	202310	Sichuan	Huidong	2605	Needle	Asymptomatic	Negative	0
C_HN14	202310	Sichuan	Huidong	2605	Needle	Asymptomatic	Negative	0
C_HN15	202310	Sichuan	Huidong	2605	Needle	Asymptomatic	Negative	0
C_HB13	202310	Sichuan	Huidong	2605	Bark	Asymptomatic	Negative	0
C_HB14	202310	Sichuan	Huidong	2605	Bark	Asymptomatic	Negative	0
C_HB15	202310	Sichuan	Huidong	2605	Bark	Asymptomatic	Negative	0
C_HN16	202310	Sichuan	Mao	2040	Needle	Asymptomatic	Negative	0
C_HN17	202310	Sichuan	Mao	2040	Needle	Asymptomatic	Negative	0
C_HB16	202310	Sichuan	Mao	2040	Bark	Asymptomatic	Negative	0
C_HB17	202310	Sichuan	Mao	2040	Bark	Asymptomatic	Negative	0
C_HN18	202310	Sichuan	Danba	1880	Needle	Asymptomatic	Negative	0
C_HN19	202310	Sichuan	Danba	1880	Needle	Asymptomatic	Negative	0
C_HB18	202310	Sichuan	Danba	1880	Bark	Asymptomatic	Negative	0
C_HB19	202310	Sichuan	Danba	1880	Bark	Asymptomatic	Negative	0
C_HN20	202310	Sichuan	Zhaojue	2521	Needle	Asymptomatic	Negative	0
C_HN21	202310	Sichuan	Zhaojue	2514	Needle	Asymptomatic	Negative	0
C_HN22	202310	Sichuan	Zhaojue	2514	Needle	Asymptomatic	Negative	0
C_HB20	202310	Sichuan	Zhaojue	2521	Bark	Asymptomatic	Negative	0
C_HB21	202310	Sichuan	Zhaojue	2514	Bark	Asymptomatic	Negative	0
C_HB22	202310	Sichuan	Zhaojue	2514	Bark	Asymptomatic	Negative	0
C_HN23	202310	Sichuan	Zhaojue	2521	Needle	Asymptomatic	Negative	0
C_HB23	202310	Sichuan	Zhaojue	2521	Bark	Asymptomatic	Negative	0
C_HN24	202310	Shaanxi	Liuba	1719	Needle	Asymptomatic	Negative	0
C_HN25	202310	Shaanxi	Liuba	1719	Needle	Asymptomatic	Negative	0
C_HB24	202310	Shaanxi	Liuba	1719	Bark	Asymptomatic	Negative	0
C_HB25	202310	Shaanxi	Liuba	1719	Bark	Asymptomatic	Negative	0
C_HN26	202310	Sichuan	Luding	2539	Needle	Asymptomatic	Negative	0
C_HB26	202310	Sichuan	Luding	2539	Bark	Asymptomatic	Negative	0
C_HN27	202310	Gansu	Liangdang	1618	Needle	Asymptomatic	Negative	0
C_HN28	202310	Gansu	Liangdang	1618	Needle	Asymptomatic	Negative	0
C_HN29	202310	Gansu	Liangdang	1618	Needle	Asymptomatic	Negative	0
C_HN30	202310	Gansu	Liangdang	1618	Needle	Asymptomatic	Negative	0
C_HN31	202310	Gansu	Liangdang	1618	Needle	Asymptomatic	Negative	0
C_HN32	202310	Gansu	Liangdang	1618	Needle	Asymptomatic	Negative	0
C_HN33	202310	Gansu	Liangdang	1618	Needle	Asymptomatic	Negative	0
C_HN34	202310	Gansu	Liangdang	1618	Needle	Asymptomatic	Negative	0
